# Theoretical design of a technetium-like alloy and its catalytic properties†
†Electronic supplementary information (ESI) available. See DOI: 10.1039/c9sc00912d


**DOI:** 10.1039/c9sc00912d

**Published:** 2019-04-26

**Authors:** Wei Xie, Michihisa Koyama

**Affiliations:** a INAMORI Frontier Research Center , Kyushu University , 744 Motooka, Nishi-ku , Fukuoka , 819-0395 , Japan . Email: xiewei@ifrc.kyushu-u.ac.jp; b Graduate School of Engineering , Hiroshima University , 1-4-1 Kagamiyama, Higashi-Hiroshima , Hiroshima 739-8527 , Japan; c Global Research Center for Environment and Energy Based on Nanomaterials Science , National Institute for Materials Science , 1-1 Namiki , Tsukuba , Ibaraki 305-0044 , Japan . Email: koyama.michihisa@nims.go.jp

## Abstract

Based on the concept of density of states (DOS) engineering, we theoretically designed a pseudo-Tc material (Mo–Ru alloy) and investigated its electronic structure, phase stability and catalytic activity by using density functional theory. Through comparing the DOS shape, peak distribution, and DOS area differences between Tc and the Mo–Ru alloy, we noticed that bcc-Mo_8_Ru_8_ and hcp-Mo_8_Ru_8_ had the most similar electronic structures to Tc. The excess energies after entropy correction of hcp-Mo_8_Ru_8_ and bcc-Mo_8_Ru_8_ are stable when the temperature is up to 765 and 745 K, respectively. These results provided the possibility of pseudo-Tc alloy (hcp-Mo_8_Ru_8_ and bcc-Mo_8_Ru_8_) synthesis. Finally, according to reaction coordinate analysis, the similar catalytic activity between hcp-Mo_8_Ru_8_ and Tc have been demonstrated in CO oxidation and N_2_ dissociation. In N_2_ dissociation, Tc has a suitable ratio of transition state (TS) barrier to reaction energy which make Tc an efficient catalyst for NH_3_ synthesis, in addition to our designed pseudo-Tc (hcp-MoRu) because of the similar electronic structures. Our finding provides valuable insight into materials and catalyst design.

## Abstract 2

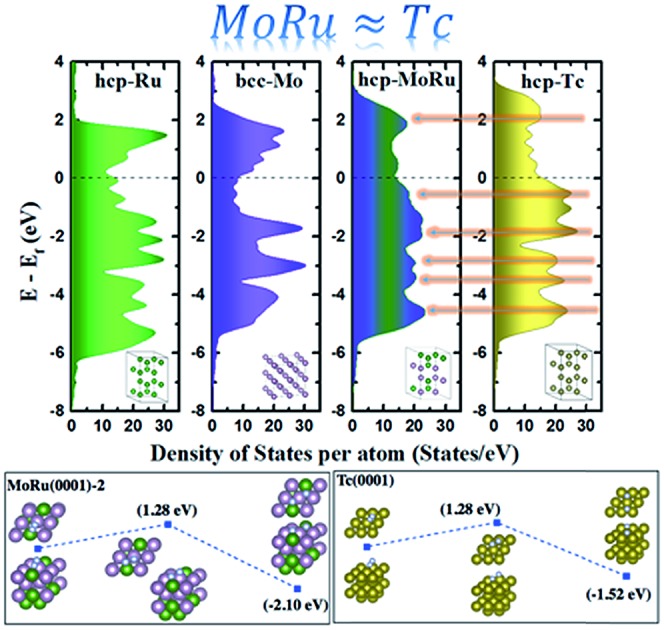
Based on the concept of density of states (DOS) engineering, we theoretically designed a pseudo-Tc material (Mo–Ru alloy) and investigated its electronic structure, phase stability and catalytic activity by using density functional theory.

## 1 Introduction

Technetium (Tc) is the lightest element among the elements for which all isotopes are radioactive. It is unstable and extremely scarce, and only minute amounts are found in the earth's crust. Technetium was discovered in 1937 by Carlo Perrier and Emilio Segrè^1^ and is named according to the Greek word for “artificial,” representing the fact that Tc is an element which is artificially made during nuclear reactions. Every year, a large amount of Tc is produced in nuclear waste. Except for the small portion used for medical applications, the disposal of Tc in nuclear waste is being taken into serious consideration. Of the greatest concern is its relatively high mobility in aqueous and geochemical environments.^2^ For safe storage, the chemical properties of Tc have been investigated.^3^ However, running chemical experiments entails considerable risk. In recent decades, a few Tc studies have been conducted, including the effect of corrosion caused by Tc on Fe^4^,^5^ and Zr^6^ in steel containers and the surface effect of Tc and its alloys in water and in an ambient environment.^3^,^4^ Technetium is more effective as a dehydrogenation catalyst than rhenium and palladium,^7^ but its radioactivity limits its study and wide application.

Practically, Tc is too dangerous to use as a commodity or for industry applications. Nonetheless, its unexplored electronic structure may lead to unique functionalities for certain applications. Recently, a new concept of density of states (DOS) engineering^8^ has been proposed as a novel approach to engineer chemical properties of alloys. DOS engineering can be used for electronic structure design for a specific application. The chemical and physical properties of elements are determined by their electronic states, which can be expressed as DOS.^9^–^12^ Based on tuning the location of the d-band center from DOS results, d-band theory has been used for catalytic design.^11^,^12^ According to the intended chemical and physical properties, the suitable DOS shape also is a key matter. By alloying every available element, we may be able to manipulate the shape of the DOS as our design to create new functional catalysts. Directly creating an original DOS for specific chemical applications is difficult; therefore, we typically start by choosing an efficient catalyst as a template when we design DOS. Some experimental studies have proved the concept of DOS engineering. A Pd_0.5_Ru_0.5_ solid-solution alloy exhibits excellent NO_*x*_ reduction activity similar to or even exceeding that of Rh.^9^ A Ag–Rh alloy exhibits hydrogen-storage properties like those of Pd.^10^ In addition, by alloying Pd with other metals, the hydrogen-storage properties of Pd can be adjusted.^13^ In the reaction of ethane formation, a metal catalyst can be replaced by transition metal carbides^14^ because the reaction barriers for the catalysts of Pd and Rh are comparable to those of Ru–C and the barrier for Ru is comparable to that for Mo–C and Tc–C. Boron also is an option that can be used to adjust the number of d electrons to create a special DOS.^15^ Although some progress in DOS engineering has been made, a scientific standard to evaluate the DOS shape between the created and the template is still lacking. In this study, we investigated four binary alloys (Fe–W, Mo–Ru, Mn–Re, and Cr–Os) with solid-solution structures to explore the possibility of creating pseudo-Tc inspired by the concept proposed by Kitagawa.^16^ All investigated elements are neighbors of Tc in the periodic table. Equal or similar compositions were adopted to ensure that the number of valence electrons in the alloys is the same as or similar to that in Tc, which has been proven to be an important factor in creating similar chemical properties.^14^,^17^,^18^ By using density functional theory (DFT), the DOS was calculated to investigate the electronic structures. Excess energy and entropy were employed to discuss the stability and possibility of synthesis. Through this study, we propose some standards to evaluate the DOS shape difference.

## 2 Computational details

All the spin-polarized calculations were performed using the Vienna *ab initio* simulation package (VASP)^19^,^20^ version 5.3.3, which is a plane-wave density functional code. The electron–electron exchange and correlation interactions were described by using the generalized gradient approximation (GGA) with the Perdew–Burke–Ernzerhof (PBE)^21^ functional form. The projector augmented-wave (PAW)^22^,^23^ method was employed to describe the interaction between the core and valence electrons. The wave functions were expanded in a plane-wave basis set with a cutoff energy of 500 eV. The convergence criterion for energy was 1 × 10^–5^ eV per cell. Monkhorst–Pack^24^ meshes of 9 × 9 × 9 *k*-point sampling in the Brillouin zone were used for bulk models and 9 × 9 × 1 k-points were used for slab models. The tetrahedron method with Blöchl corrections^23^ was employed to run an accurate total energy calculation. Both hcp and bcc phases of bulk structures were considered for these alloys because Mo, Fe, W, Cr, and Mn are bcc metals and Ru, Os and Re are hcp metals (the optimized models of the bulk are shown in Fig. S1 and S2,† as well as their XRD patterns calculated using VESTA). Here a Mo–Ru alloy is taken as an example. For the slab models, we cleave the (0001) and (100) surfaces of hcp and bcc metals because they are the predominant growth surfaces. The vacuum layer was set at about 15 Å. The transition states were obtained by using the Climbing Image-Nudged Elastic Band method.^25^

## 3 Results and discussion

The chemical and physical properties of these alloys are determined by their electronic structures, which are typically represented as the DOS. The shape, intensity, and band width, the contribution of s, p, d, and f orbitals of the DOS, and the valence and conduction levels are all the basic factors that affect the optical, magnetic, and thermodynamic properties.^9^ To construct pseudo-Tc, an electronic structure analogous to that of Tc is required. Therefore, after optimization, we first calculated the DOS to evaluate the electronic structures of alloys. Fig. S3† shows the DOS of Mo–Ru alloys with different mixture ratios. bcc-Mo_8_Ru_8_ and hcp-Mo_8_Ru_8_ have the same valence band width as Tc and their main DOS peaks are located in a similar energy level to those of Tc, which indicates the similar electronic structure. However, for the other ratios, large differences exist between alloys and Tc, which indicated that the same valence electron number is just one of the factors important for creating a similar electronic state. Furthermore, we also investigated the DOS of different kinds of alloys (Fe–W, Os–Cr, Mn–Re, and Mo–Ru alloys). The number of electrons in these alloys is the same as that in Tc. In Fig. S4,† there are wide variations, which proved that having the same orbital state is an important factor. For Mo–Ru alloys, the valence DOS resulted from 4d–4d orbital hybridization, while for other alloys, the nature of the DOS resulted from 3d–5d orbital hybridization.

**Fig. 1:**
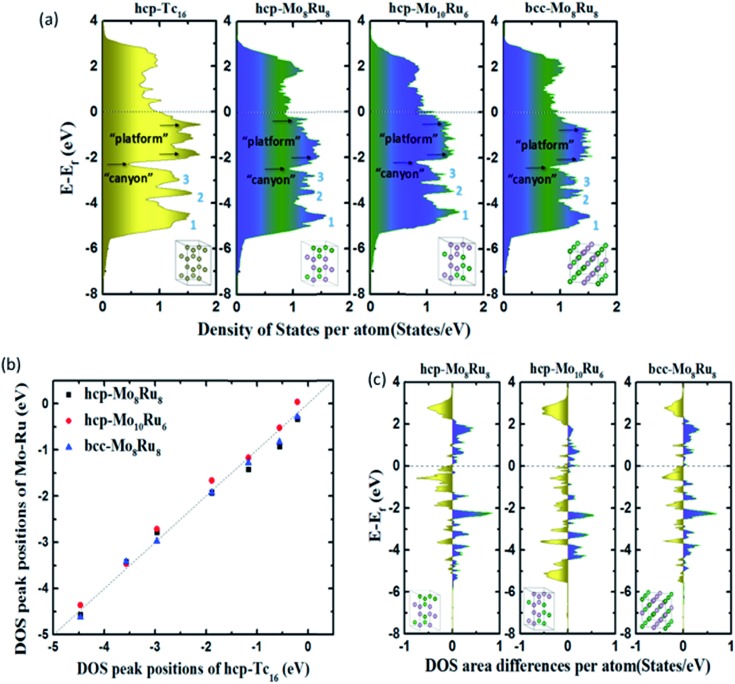
(a) Total DOS, (b) typical DOS peak positions, and (c) DOS integral differences of hcp-Mo_8_Ru_8_, hcp-Mo_10_Ru_6_, and bcc-Mo_8_Ru_8_ compared with that of hcp-Tc_16_.

Detailed analysis on hcp-Mo_8_Ru_8_, hcp-Mo_10_Ru_6_, and bcc-Mo_8_Ru_8_ has been carried out. We investigated the positions and intensities of specific peaks in the DOS, the band width, and the DOS area difference to evaluate the electronic structures. Some of these terms have already been used to evaluate the electronic structures and properties.^8^,^9^ Fig. 1(a) shows the total DOS of hcp-Tc_16_, hcp-Mo_8_Ru_8_, hcp-Mo_10_Ru_6_, and bcc-Mo_8_Ru_8_. We separated the whole energy level into three parts with boundaries being marked as “canyons” (located at –2.31, –2.47, –2.16, and –2.42 eV for hcp-Tc_16_, hcp-Mo_8_Ru_8_, hcp-Mo_10_Ru_6_, and bcc-Mo_8_Ru_8_, respectively) and the Fermi level. Fig. 1(b) shows the shift of DOS peak positions among hcp-Mo_8_Ru_8_, bcc-Mo_8_Ru_8_, and hcp-Mo_10_Ru_6_, with respect to hcp-Tc_16_. In the low-energy-level range from negative infinity to the “canyon” boundary, three of the stronger DOS intensity areas can be easily identified (numbered as peaks 1, 2, and 3) in Fig. 1(a). All three alloys have similar peak positions corresponding to those of hcp-Tc_16_. The peak position differences of hcp-Mo_8_Ru_8_, hcp-Mo_10_Ru_6_, and bcc-Mo_8_Ru_8_ from those of hcp-Tc_16_ are very small, with the largest difference being 0.28 eV (for hcp-Mo_10_Ru_6_, as given in Table S1†). The intensity differences between the identified peaks are also small and the largest difference is 0.20 states per eV per atom (for hcp-Mo_8_Ru_8_, as given in Table S1†). Here we only compare the position of the DOS peak center and the peak intensity because, with these two terms, we can ascertain that the valence electrons are located at a similar energy level and can estimate how many electrons are at this energy level. In the middle part of the range from the “canyon” boundary to the Fermi level, a greater DOS difference between the Mo–Ru alloy and Tc can be observed. The electrons in orbitals near the Fermi level are more active, being sensitive to the environment, which leads to DOS shape changes. In the DOS of hcp-Tc_16_, two single peaks and one split strong peak at –1.89, –1.17, and –0.56 eV can be clearly identified. However, in the case of the alloy, the DOS curves become more complex and it becomes difficult to differentiate the stronger intensity point from the others. For hcp-Mo_8_Ru_8_, the two split DOS peaks (–1.30 and –1.95 eV) correspond with the DOS peaks (at *ca.* –1.10 and –1.90 eV) in hcp-Tc_16_. These is no DOS peak in hcp-Mo_8_Ru_8_ that can match the peak located at –0.56 eV in hcp-Tc_16_, but some weak (noise-like) peaks are present in this range. For hcp-Mo_10_Ru_6_ and bcc-Mo_8_Ru_8_, from the “canyon” boundary to the Fermi level, the DOS shape is a broad platform. These DOS shape changes are caused by the formation of *n*-fold degenerate orbitals during alloying. Geometrically, for hcp-Tc_16_, there are only Tc–Tc bonds, thus resulting in the same bond length and hybrid orbitals. For alloy systems, there are three kinds of bonds (Ru–Ru, Ru–Mo, and Mo–Mo), and the random arrangement of atoms leads to different bonds lengths. Electronically, for hcp-Tc_16_, high symmetry means that the valence electrons are in the same electronic environment, so the electrons are localized in several specific energy levels, which leads to the stronger DOS peaks. For alloy systems, the lower symmetry and irregular electron distribution result in the broader DOS peaks. Therefore, according to this situation, we used a range of platform energies and the average intensity of the DOS to discuss their similarity. The energy range of the platform is shown in Fig. 1(a); the start and end positions were obtained from the local maximum DOS points and the values are listed in Table S2.† The position differences of the platform and DOS intensities between alloys and hcp-Tc_16_ are less than 0.3 and 0.2 states per eV per atom, respectively. However, the choice of start and end positions and the calculation of the DOS intensity are not accurate enough because of the complex DOS shape.

DOS area differences are represented by the DOS integral differences, and the integral of the differences was calculated by using the equation 
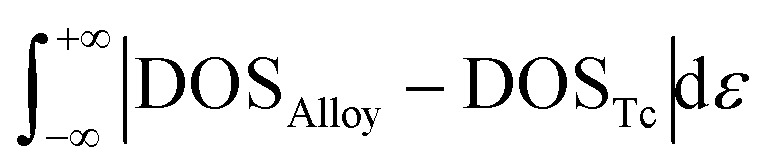
. The DOS area differences between Mo–Ru alloys and Tc are shown in Fig. 1(c) and the different integral values (absolute values) are listed in Table 1. At the lower energy level from negative infinity to –2.3 eV (the largest positive integral differences), the area difference in hcp-Mo_10_Ru_6_ is much larger than that in hcp-Mo_10_Ru_6_ and bcc-Mo_8_Ru_8_. In the higher energy level range from –2.3 eV to the Fermi level, the area difference of hcp-Mo_10_Ru_6_ becomes smaller. In Fig. 1(c), for hcp-Mo_8_Ru_8_ near the Fermi level, the large area difference is caused by the platform. From the DOS of hcp-Tc_16_ in Fig. 1(a), we noticed a strong DOS peak (at –0.56 eV) near the Fermi level, while for the alloys it becomes weak. The integral differences in this local range follow the order of hcp-Mo_10_Ru_6_ < bcc-Mo_8_Ru_8_ < hcp-Mo_8_Ru_8_. hcp-Mo_10_Ru_6_ is the most promising candidate in the local range from –2.3 eV to the Fermi level. However, for the whole energy level, a different conclusion is reached. Table 1 lists the values of DOS area differences; bcc-Mo_8_Ru_8_ has the smallest area differences with a value of 1.36 per atom for the whole energy level and the order is bcc-Mo_8_Ru_8_ < hcp-Mo_8_Ru_8_ (1.59 states per eV per atom) < hcp-Mo_10_Ru_6_ (1.63 states per eV per atom). These results agree with the conclusion that iso-valence electrons are one of the important factors in creating similar electronic structures. Finally, we compared the band widths of hcp-Mo_8_Ru_8_, hcp-Mo_10_Ru_6_, bcc-Mo_8_Ru_8_ and hcp-Tc_16_ listed in Table 1. For this term, the three alloys have a new order. hcp-Mo_8_Ru_8_ has almost the same d-band width as hcp-Tc_16_ (with a difference of 0.02 eV). The largest width difference is from bcc-Mo_8_Ru_8_ with a value of 0.48 eV. In addition, we also calculated the d-band center (listed in Table 1) as a factor for comparison. Based on the analysis above, all three alloys have the potential to act as pseudo-Tc, because each of them has respective similar features to those of Tc. bcc-Mo_8_Ru_8_ and hcp-Mo_8_Ru_8_ are evaluated as having more features that are similar to those of Tc, making them the first and second pseudo-Tc candidates.

**Table 1:** Integral differences per atom, d-band width, and the d-band center

	Integral differences	d-band width (eV)	Start (eV)	End (eV)	d-band center (eV)
hcp-Tc_16_	0	11.58	–6.57	5.01	–1.33
hcp-Mo_8_Ru_8_	1.59	11.60	–7.02	4.58	–1.48
hcp-Mo_10_Ru_6_	1.63	11.15	–6.81	4.33	–1.44
bcc-Mo_8_Ru_8_	1.36	11.10	–6.82	4.28	–1.42

**Fig. 2:**
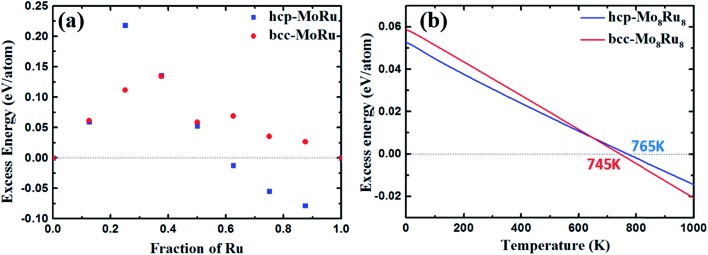
Excess energy of Mo–Ru alloys with both the hcp and bcc phase: (a) excess energy and (b) excess energy after entropy correction.

According to the electronic structure analysis above, hcp-Mo_8_Ru_8_ and bcc-Mo_8_Ru_8_ both have the potential to be pseudo-Tc because of their highly similar DOS shapes. However, according to the phase diagram^26^,^27^ of Mo–Ru alloys, in our suggested ratio, Mo–Ru alloys exists as phase-separated structures. A solid-solution structure is metastable. The excess energies^28^ are calculated and displayed in Fig. 2(a) to evaluate the phase stability and possibility for synthesis. The calculated excess energies exhibit the same tendency as seen in the phase diagram^26^,^27^ and as noted in other reports.^29^ It is obvious that the hcp type is the most stable phase when the Ru ratio is >60% because, in this Ru ratio range, negative excess energy is reported in this study and this has been experimentally proved.^27^ The bcc types of Mo–Ru alloys are unstable in the whole Ru ratio range because of the positive excess energy. In Fig. 2(a), we also noticed that bcc-Mo_14_Ru_2_ and hcp-Mo_14_Ru_2_, and bcc-Mo_8_Ru_8_ and hcp-Mo_8_Ru_8_ have very similar excess energies. By comparing their XRD patterns shown in Fig. S1 and S2,† we found that after optimization, hcp-Mo_14_Ru_2_ transformed into the bcc type, and bcc-Mo_8_Ru_8_ transformed into the hcp type, which indicated that with solid-solution structures, Mo–Ru alloys in these ratios had only one stable phase. For Mo–Ru alloys with a high Ru ratio such as Mo_6_Ru_10_, Mo_4_Ru_12_, and Mo_2_Ru_14_, their excess energies split, and the XRD patterns suggested that bcc-Mo_6_Ru_10_, bcc-Mo_4_Ru_12_, and bcc-Mo_2_Ru_14_ had transformed into the fcc phase, while hcp-Mo_6_Ru_10_, hcp-Mo_4_Ru_12_, and hcp-Mo_2_Ru_14_ remained in the hcp phase. The existence of both the hcp and fcc phase for Ru provides a good explanation for this result. The fcc phase of Ru as the stable structure for Ru nanoparticles has been discovered and synthesized.^30^ The stability of its alloy in the fcc phase has also been evaluated in experimental^31^ and theoretical studies.^32^ The phase of Mo–Ru alloys with high Ru ratio transforms from bcc to fcc instead of hcp because of the similarity of the electronic structure between the bcc and fcc phase. For the bcc and fcc phase, the t_2g_ and e_g_ orbitals are separated, the d_*xy*_, d_*yz*_, and d_*xz*_ orbitals are highly hybridized leading to the same DOS shape in the crystal. The same results are seen for the orbitals of d_*x*^2^–*y*^2^_ and d_*z*^2^_. However for the hcp phase, the t_2g_ orbitals are separated, the orbitals of d_*xz*_ and d_*yz*_ are located on the same energy level, and the orbitals of d_*xy*_ and d_*x*^2^–*y*^2^_ are located on the same energy level, while the orbital of d_*z*^2^_ is located on a different energy level. In our suggested alloy range, neither the hcp phase (hcp-Mo_8_Ru_8_) nor the bcc phase (bcc-Mo_8_Ru_8_) is stable because of the positive excess energy. They are thermodynamically unstable. However, the excess energies are very small, with values of 0.052 and 0.059 eV per atom for hcp-Mo_8_Ru_8_ and bcc-Mo_8_Ru_8_, respectively.

The effect of entropy on excess energy^33^ was considered to discuss the relative stabilities of hcp-Mo_8_Ru_8_ and bcc-Mo_8_Ru_8_ at finite temperatures. The entropy here includes configurational and vibrational entropies. In our random solid-solution models, we fully considered the effect of symmetry in the bulk system to reduce the possible configurations in hcp and bcc phases. The configurational entropy reaches a maximum of 5.97 × 10^–5^ eV per K per atom, which is similar to the published data for binary alloys.^33^,^34^ For the phase-separated structure, the number of possible structures is unity. The effect of vibrational entropy (Table S3†) is very small compared with that of configurational entropy (5.97 × 10^–5^ eV per K per atom) after subtracting the part from the phase-separated structure. Even when the temperature reaches 1000 K, the thermodynamic effect of vibrational entropy is 7.28 × 10^–6^ eV per K per atom for hcp-Mo_8_Ru_8_, while it is 1.96 × 10^–5^ eV per K per atom for bcc-Mo_8_Ru_8_. That is, increasing configurational entropy is one of most efficient methods for creating a phase-stable alloy.

Fig. 2(b) shows the excess energy after entropy correction. In the temperature range from 0 to 765 K for hcp-Mo_8_Ru_8_ and to 745 K for bcc-Mo_8_Ru_8_, the Mo–Ru alloys are still unstable because of the positive values. However, the excess energies keep decreasing as the temperature increases. When the temperatures are up to 765 K for hcp-Mo_8_Ru_8_ and up to 745 K for bcc-Mo_8_Ru_8_, solid-solution Mo–Ru alloys can be stable and synthesized. Furthermore, depending on the synthesis conditions, hcp and bcc can be retained up to a certain range of temperatures.^35^ That is, when hcp-Mo_8_Ru_8_ and bcc-Mo_8_Ru_8_ are formed by cooling, they may be stable in a lower temperature range.^34^ These excess energy results suggest that solid-solution Mo_8_Ru_8_ for both bcc and hcp types can be synthesized and stable when the temperatures are up to 745 and 765 K, respectively. When combined in the form of nanoparticles, Mo–Ru can be expected to be synthesized at a lower temperature because of its large specific surface area, which gives the surface energy a larger weightage in the total energy system of the alloy.^36^,^37^

The basic idea for catalyst design in our study is that the catalytic activities fundamentally originate from the electronic structures presented as DOS; creating a similar electronic structure (DOS) leads to the similar catalytic process in chemistry. In order to validate our idea, we simulated the surface reactions of CO oxidation and N_2_ dissociation for NH_3_ synthesis over monometals (Tc, Mo, and Ru) and Mo–Ru alloys. Up to now there has been no study on Tc in catalytic applications, while plenty of studies for its neighboring metals (Ru and Mo) can be found.^38^–^43^ From these studies, we can obtain some insights as follows. Ru (Ru–Cu,^38^ Ru–Pt,^39^ and Ru–O^40^ for CO oxidation and Ru–Ba^41^ for NH_3_ synthesis) and Mo (Rh/SiO_2_/Mo^42^ for CO and a Mo cluster^43^ for N_2_ dissociation) based catalysts exhibit good activity toward CO oxidation and NH_3_ synthesis. However, neither monometal Ru nor Mo is an efficient catalyst for these reactions. Slight modifications may lead to their enhanced catalytic activity. On the other hand, in NH_3_ synthesis, compared to Mo and Ru, Tc may be located in hot-spot areas of steady-state TOF^44^ according to the position of Tc in the periodic table. For hcp-Mo_8_Ru_8_, we constructed four slab models as shown in Fig. S5† to investigate all the possible sites in hcp-Mo_8_Ru_8_ considered. Here we did not cleave the surface based on bcc-Mo_8_Ru_8_, because according to the stability analysis above, bcc-Mo_8_Ru_8_ transformed into the hcp phase (Fig. S1†). In order to make sure that our approach also works for the partial density of states and local density of states, we evaluated the DOS shape similarity of the alloy surface to that of Tc(0001), as well as the DOS of local active sites. Fig. S6 and S7† show the DOS area differences, and Table 2 lists the values of the d-band center and DOS area integral differences. After alloying, the d-band centers of four MoRu(0001) surface models are –1.70 eV, –1.32 eV, –1.29 eV, and –1.47 eV, which are close to the –1.40 eV of the Tc surface, and different from those of Ru(0001) (–1.90 eV) and Mo(100) (–0.99 eV), except for MoRu(0001)-1, which is a Ru-segregated surface model. The active sites on Mo–Ru alloys show a more similar electronic structure with the d-band center values of –1.39 eV, –1.38 eV, and –1.39 eV for MoRu(0001)-2, MoRu(0001)-3, and MoRu(0001)-4, respectively. The DOS area differences obtained for these three surfaces are confirmed to be small from Fig. S6 and S7† and Table 2. Both the d-band center and DOS area difference suggest that the local electronic state of Tc has been reproduced by alloying Mo–Ru. Next, we simulated the catalytic reactions on the surfaces.

**Table 2:** The d-band center and the DOS integral differences of the surface (S) and active site (AS) for each model. The active site for Mo(100) is the hcp site of Mo_4_; the active sites for Tc(0001), Ru(0001), MoRu(0001)-1 are the fcc/hcp sites of Tc_3_, Ru_3_, and Ru_3_; the active sites for MoRu(0001)-2, MoRu(0001)-3, and MoRu(0001)-4 are the fcc/hcp sites of Mo_2_Ru

S/AS	d-band center (eV)	Integral differences
Tc(0001) S(AS)	–1.40	0
Ru(0001) S(AS)	–1.90	5.49
Mo(100) S(AS)	–0.99	4.75
MoRu(0001)-1 S(AS)	–1.70	3.06
MoRu(0001)-2 S	–1.32	2.99
MoRu(0001)-2 AS	–1.39	2.83
MoRu(0001)-3 S	–1.29	2.45
MoRu(0001)-3 AS	–1.38	2.38
MoRu(0001)-4 S	–1.47	2.99
MoRu(0001)-4 AS	–1.39	2.99

CO oxidation on the catalyst surface is usually accompanied by adsorption as the initial process. The fcc, top and 4 fold-hollow sites are the most stable sites for CO adsorption on Tc(0001), Ru(0001), and Mo(100) with values of –1.80 eV, –1.91 eV, and –2.21 eV as shown in Table S4† (adsorption structures are also shown in Table S4†), respectively. However, the adsorption structure of CO on the top sites is crucially important for oxidation.^45^,^46^ On the Tc(0001) surface, the calculated CO adsorption energy on the top sites is the highest (–1.72 eV compared to –1.91 eV and –1.73 eV on the top sites of Ru(0001) and Mo(100), respectively). Table S5† summarizes the adsorption structures and energies of CO on four MoRu(0001) surfaces. The adsorption energies for CO on MoRu(0001) facilitate bipolar distribution between the Ru-top site (–1.95 to –2.08 eV) and Mo-top site (–1.59 to –1.73 eV). After alloying, more electrons distribute on Ru potentially enhancing the electron back-donation capability, which will lead to stronger CO adsorption. Contrary to CO adsorption, O adsorption favors 3-fold sites including Mo. The most stable sites are Mo_3_-3 fold hollow sites (less than –4.00 eV as shown in Table S6†). The MoRu_2_-3 fold hollow sites have the most similar adsorption energies to those of Tc (–3.43 eV and –3.01 eV of MoRu_2_-hcp and MoRu_2_-fcc on MoRu(0001)-4 compared to the –3.67 eV and –3.01 eV of Tc_3_-hcp and Tc_3_-fcc shown in Table S4†).

**Fig. 3:**
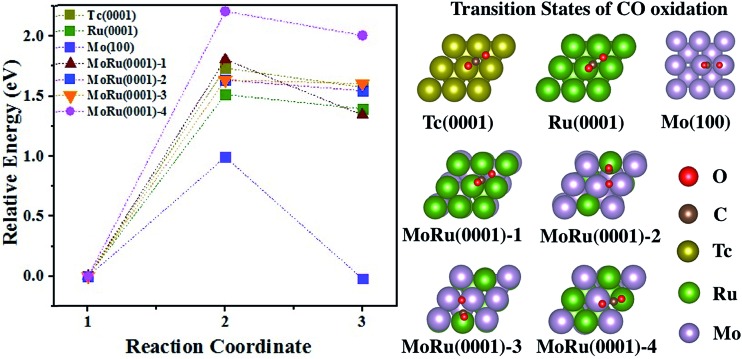
The energy pathway for the CO oxidation process (CO* + O* → CO_2_*) on Tc(0001), Ru(0001), Mo(100), and four MoRu(0001) surfaces. The calculated energy pathway of CO oxidation and the structures of the TS for CO oxidations. The IS, TS, and FS structures are shown in Fig. S8.†

The catalytic processes of CO oxidation over Tc(0001), Ru(0001), Mo(100), and MoRu(0001) are summarized in Fig. 3 (the initial state (IS), transition state (TS), and final state (FS) are shown in Fig. S8†). The analysis of adsorption structures above suggests that the molecular CO is favorable on Ru-top sites while the atomic O is favorable on Mo_3_-3 fold-hollow sites. MoRu(0001) has both kinds of adsorption sites, and these sites reduce the competitive adsorption by separately adsorbing molecular CO and atomic O, leading to the most stable co-adsorption (shown in Table 3). This indicates that CO oxidation will follow the Langmuir–Hinshelwood (LH) model on MoRu(0001). In order to investigate the processes of different electronic structure driven reactions, the LH model was employed for CO oxidation on all the surface models. The calculated activation energy of CO_(ad)_ + O_(ad)_ → CO_2(ad)_ on Tc(0001) is 1.73 eV, higher than that on Ru(0001) and Mo(100) with values of 1.51 eV and 0.99 eV. Mo(100) executes a different reaction process due to the totally different morphology. For Ru(0001), the lower activation energy is due to the weak O adsorption energy (–2.90 eV compared with –3.67 eV on Tc(0001)). The MoRu(0001) surfaces have activation energies with values of 1.80 eV, 1.64 eV, 1.64 eV, and 2.20 eV for the four slab models, respectively, as shown in Table 3. The activation energies distribute around the value for Tc, *i.e.* 1.73 eV, and we can conclude that Mo–Ru will show catalytic properties similar to those of Tc. These energy deviations on MoRu(0001) were due to the randomness of solid-solution structures. Unlike monometals, there are many different catalytic sites for CO oxidation, which extend the energy range with a certain average value similar to that for Tc. These results also confirmed DOS analysis that the DOS shape of MoRu alloys is moderate compared with that of hcp-Tc_16_. It should be noticed that Mo is determined to be the best catalyst for CO oxidation because of the lowest activation energy (0.99 eV) by assuming the same LH mechanism just for a simple comparison of different catalysts. However, much stronger O adsorption (–4.08 eV) than CO (–2.21 eV) on the same hollow sites of Mo(100) will make Mo surface oxidized, instead of yielding the co-adsorption structure, which will lead to different mechanisms such as the Eley–Rideal mechanism. This will make Mo a poor catalyst for CO oxidation in actual experiments.

**Table 3:** Activation energy (*E*_a_) and reaction energy (*E*_r_) of CO oxidation, and co-adsorption energy (*E*_Co-ads_) of molecular CO and atomic O on these surfaces 
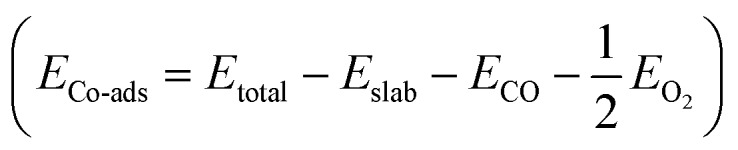

Surface	*E*_a_ (eV)	*E*_r_ (eV)	*E*_Co-ads_ (eV)
Tc(0001)	1.73	1.57	–5.22
Ru(0001)	1.51	1.39	–4.73
Mo(100)	0.99	–0.02	–4.97
MoRu(0001)-1	1.80	1.34	–4.68
MoRu(0001)-2	1.63	1.54	–5.91
MoRu(0001)-3	1.63	1.60	–5.86
MoRu(0001)-4	2.20	2.00	–5.92

**Fig. 4:**
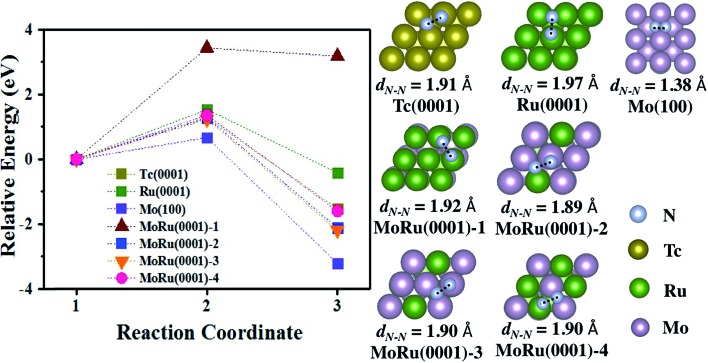
The energy pathway for the N_2_ dissociation process (N_2_* → 2N*) on Tc(0001), Ru(0001), Mo(100), and four MoRu(0001) surfaces. The calculated energy pathway of N_2_ dissociation and the structures of the TS for N_2_ dissociation. The IS, TS, and FS are shown in Fig. S9.†

We next investigated the catalytic activity for NH_3_ synthesis. For the NH_3_ synthesis process, some experimental and theoretical studies have identified N_2_ dissociation as the rate-determining step.^47^,^48^ Furthermore, it is reported that very weak N_2_ adsorption energies and very strong binding of intermediates (such as *N) will also prevent the reaction process.^44^,^49^ Therefore, the best catalyst will have an ideal balance between the activation energy (*E*_a_) and adsorption energy of *N and N_2_ (evaluated using the reaction energy (*E*_r_)), which is located in a suitable area described by Bell–Evans–Polanyi or Brønsted–Evans–Polanyi scaling relationship. In Aayush's study,^44^ Mo and Ru do not exhibit outstanding catalytic activities, which suffer from the very strong N adsorption interaction (Mo metal), and high N_2_ dissociation activation energy (Ru metal), respectively. Because Tc is located between Mo and Ru in the periodic table, it is expected to be an excellent catalyst for NH_3_ synthesis, as well as our designed pseudo-Tc materials (Mo–Ru alloys). As shown in Table S7,† N_2_ is adsorbed at bridge sites and the adsorption energies are –0.91 eV, –0.32 eV, and 0.07 eV on Mo(100), Tc(0001), and Ru(0001), respectively. Also for N_2_ on MoRu(0001), the bridge site is a favorable site as shown in Table S7.† The adsorption energies are distributed in the range from –0.36 eV to –0.89 eV, which is due to non-uniform adsorption sites of the MoRu(0001) surface. The energy profile of N_2_ dissociation on these surfaces are shown in Fig. 4, and the corresponding key properties including the activation energy and reaction energy are summarized in Table 4. As reported,^44^ Mo has a low N_2_ activation energy (0.67 eV) and reaction energy (–3.20 eV), suffering from the strong *N binding energy. The activation energy is as large as 1.53 eV, indicating that N_2_ is not easily dissociated at room temperature. The activation energy on Tc is 1.28 eV while the reaction energy is –1.53 eV, which is close to the suitable area of steady-state TOF in NH_3_ synthesis. Following our design, Mo–Ru alloys exhibit similar catalytic activity to Tc in N_2_ dissociation. From Table 4, on the MoRu(0001) surface, the activation energies for N_2_ dissociation are concentrated in narrow range (1.23 eV to 1.36 eV), close to the 1.28 eV of that on Tc(0001). The structures of the TS shown in Fig. 4 on MoRu(0001) are similar to that on Tc (one N atom is on the top-site and the other N is on the bridge-site in the opposite direction). The elongated N–N distances in the TS structure on Mo–Ru surfaces are 1.89 Å, 1.90 Å, and 1.90 Å, which are close to the N–N distance of 1.91 Å on Tc(0001). This indicates that the process of N_2_ dissociation on Mo–Ru is similar to that on Tc(0001), and the Mo_0.5_–Ru_0.5_ alloy reproduced the chemical properties of Tc in our design. Also, we noticed one exception of MoRu(0001)-1 (Ru-skin); N_2_ dissociation on this surface suffers from the highest activation energy with a value of 3.43 eV. This is because the reaction proceeds in a different manner. In Table S7,† for MoRu(0001)-1, N_2_ is adsorbed on the top sites with an end-on configuration while tilted end-on or side-on adsorptions are observed on other surfaces. Before forming the most stable N-hollow adsorption structures, two dissociated N atoms must be first formed from the straight end-on configuration, which leads to a higher reaction energy (3.18 eV). MoRu(0001)-1 is understood to be an extreme example of core–shell and phase-separated structures in alloy catalyst structures.

**Table 4:** The activation energy (*E*_a_) and reaction energy (*E*_r_) for N_2_ dissociation, and adsorption energy (*E*_ads-N_2__) of molecular N_2_(*E*_ads-N_2__ = *E*_total_ – *E*_slab_ – *E*_N_2__), *E*_ads-N_2__ listed here was calculated by using the IS

Surface	*E*_a_ (eV)	*E*_r_ (eV)	*E*_ads-N_2__ (eV)
Tc(0001)	1.28	–1.52	–0.32
Ru(0001)	1.54	–0.41	0.07
Mo(100)	0.67	–3.20	–0.91
MoRu(0001)-1	3.43	3.18	–0.49
MoRu(0001)-2	1.28	–2.10	–0.62
MoRu(0001)-3	1.23	–2.19	–0.69
MoRu(0001)-4	1.36	–1.59	–0.39

## 4 Conclusions

In summary, we theoretically designed a new pseudo-Tc material of Mo–Ru alloys and investigated its electronic structure and phase stability. From the DOS analysis, hcp-Mo_10_Ru_6_, hcp-Mo_8_Ru_8_, and bcc-Mo_8_Ru_8_ were identified as suitable candidates of pseudo-Tc because they have DOS shapes and peak distributions similar to those of Tc throughout the whole energy level. The DOS area differences from those of Tc follow the order of bcc-Mo_8_Ru_8_ < hcp-Mo_8_Ru_8_ < hcp-Mo_10_Ru_6_. In the suggested Ru ratio range, neither hcp-Mo_8_Ru_8_ nor bcc-Mo_8_Ru_8_ is stable because of the positive excess energy; however, the values are small (0.052 eV per atom for hcp-Mo_8_Ru_8_ and 0.059 eV per atom for bcc-Mo_8_Ru_8_). The effect of entropy was investigated to evaluate the stability at finite temperatures, especially configurational entropy. After entropy correction, hcp-Mo_8_Ru_8_ and bcc-Mo_8_Ru_8_ are stable when the temperature is up to 765 and 745 K, respectively. This result agrees well with the preceding study for bulk Mo–Ru.^25^ However, the excess energy and high-temperature range also led to the limitations of using the bulk form in synthesis, while using nanoparticles may be a breakthrough for synthesizing Mo–Ru alloys in the low-temperature range because of the larger contribution of the surface energy to the energy of the whole system. In a reaction simulation of CO oxidation and N_2_ dissociation, MoRu(0001) has related energies (adsorption energy, activation energy, and reaction energy), reaction process and related structures (IS, TS, and FS) similar to those of Tc(0001). These results indicate that our designed solid-solution Mo_0.5_Ru_0.5_ alloy can reproduce the chemical properties of Tc. The existence of exceptions (RuMo(0001)-1, and Ru-skin) proved the importance of the local site structure, revealing the limitation of core–shell and phase-separated structures in catalyst design. Our results on electronic structure and phase stability have important implications in materials design for catalysts, particularly for binary alloys.

## Conflicts of interest

There are no conflicts to declare.

## Supplementary Material

Supplementary information
