# Supplementary material for: Theoretical design of a technetium-like alloy and its catalytic properties
Source: Chem Sci. 2019 Apr 26;10(21):5461–9. doi: 10.1039/c9sc00912d (PMC6566293; doi:10.1039/c9sc00912d)
Supplement: Supplementary file 1 [file SC-010-C9SC00912D-s001.pdf]

Electronic Supplementary Material (ESI) for Chemical Science.
This journal is © The Royal Society of Chemistry 2019

Supporting Information

Theoretical design for a Technetium-like Alloy and its catalytic Properties.

Wei Xie[a, *], Michihisa Koyama[a, b ,c, *]

[*] Corresponding authors

[a] INAMORI Frontier Research Center, Kyushu University, 744 Motooka, Nishi-ku, Fukuoka, 819-0395, Japan

E-mail: xiewei@ifrc.kyushu-u.ac.jp

[b] Graduate School of Engineering, Hiroshima University, 1-4-1 Kagamiyama, Higashi-Hiroshima, Hiroshima 739-8527, Japan

[c] Global Research Center for Environment and Energy based on Nanomaterials Science, National Institute for Materials Science, 1-1 Namiki, Tsukuba, Ibaraki 305-0044, Japan

E-mail: koyama.michihisa@nims.go.jp

Computation details

All the spin-polarized calculations were performed using the Vienna ab initio simulation package (VASP) [1,2] version 5.3.3, which is a plane-wave density functional code. The electron–electron exchange and correlation interactions were described by using the generalized gradient approximation (GGA)[1] with the Perdew–Burke–Ernzerhof (PBE)[3] functional form. The projector augmented-wave (PAW)[4,5] method was employed to describe the interaction between the core and valence electrons. The wave functions were expanded in a plane-wave basis with a cutoff energy of 500 eV. The convergence criteria included threshold values of 1×10^{-5} eV for energy. Monkhorst Pack[6] meshes of 9 × 9 × 9 *k*-point sampling in the bulk Brillouin zone were used for bulk models and 9 × 9 × 1was used for slab models. A tetrahedron method with Blöchl corrections[5] was employed to run an accurate total energy calculation. The calculation models are shown in Figure.S1, with Mo–Ru alloy taken as an example. Given that bulk structures of Fe($3d^6 4s^2$), Mn($3d^5 4s^2$), W($5d^4 6s^2$), Mo($4d^5 5s^1$), and Cr($3d^5 4s^1$) are bcc types, while structures of Os($5d^6 6s^2$), Ru($4d^7 5s^1$), and Re($5d^5 6s^2$) are hcp types, we modeled both hcp and bcc structures for these alloys with a 2 × 2 × 2 unit cell including 16 atoms. The 4-layer slab models were constructed based on the optimized bulk structures (shown in Figure S5). The bottom two layers were fixed while the rest layers were relaxed. (0001) for hcp types and (100) for types have been employed for our calculations because they are ones of predominant growth surfaces.

In general, phase-separated, intermetallic, and solid-solution structures are the major alloy configurations. Phase-separated and intermetallic structures are not suitable for

designing chemical properties as pseudo-elements while the solid-solution structure has been proven to be more suitable. To confirm the randomness of the arrangement of elements in alloys, we employed the Warren–Cowley parameter[7], which is an index to evaluate short-range order. The Warren–Cowley parameters (a_i) of our bulk models were calculated according to the following formula:

$$a_i = 1 - \frac{P_A^i}{C_A}$$

where, for a binary A–B alloy, P_A^i is the conditional probability of having B atoms as neighbors in the ith coordination sphere substrate and C_A is the concentration of A atoms. When a_i is close to 0, the distribution of A and B were complete random in the alloy (solid solution). When a_i < 0, A–B bonds were dominant in the alloy (local order or supercell) while, when a_i > 0, A–A bonds were dominant in the alloy (phase-separated or clustering). The homogeneousness of the models shown in Figure 1 are checked in terms of a_i. All of these models have a small value close to 0 or equal to 0, and so they can be treated as solid solutions.

To evaluate the phase stability and possibility for synthesis, excess energy[8] was calculated. The two Mo–Ru alloys and the referred hcp-Ru and bcc-Mo were built with the same numbers of atoms, so that the calculated excess energy can be comparable. The excess energy was calculated by using the equation

$$E_{excess} = \frac{1}{16}\left(E_{Ru-Mo} - \frac{x}{16}E_{Ru} - \frac{16-x}{16}E_{Mo}\right),$$

where E_{Ru-Mo} is the total energy of the Mo–Ru alloy system, E_{Ru} is the total energy of the hcp-Ru_{16} structure, E_{Mo} is the total energy of the bcc-Mo_{16} structure, and x is the number of Ru atoms in one cell of the Mo–Ru alloy.

The entropy-corrected excess energy[9] was calculated by using the equation

$$E = E_{excess} - TS,$$

where E is the relative energy after the entropy correction, E_{excess} is the excess energy, T is the temperature, and S is the entropy. The entropy S is total entropy, which includes the vibrational entropy S_{vib} and the configurational entropy S_{conf}. The configurational entropy S_{conf} was calculated by using the following equation:

$$S_{conf} = k_B \ln w,$$

where w is the number of possible configurations and k_B is Boltzmann's constant. In our hcp-Mo_8Ru_8 of random solid solution, we fully considered the symmetry effect in the bulk system to reduce the possible configurations in hcp and bcc phases; the configuration entropy reaches a maximum of 5.97×10^{-5} eV/K per atom, which is similar to the published data for binary alloys[9,10]. For the phase-separated structure, the number of possible structures is one.

The vibrational entropy S_{vib} is determined by solving the following equation using PHONOPY version 1.11.6.20:

$$S_{vib} = -\frac{1}{2\mathrm{T}}\sum_{qv} hw(qv)\coth\left(\frac{hw(qv)}{2k_BT}\right) - k_B\sum_{qv}\ln 2\sin\left(\frac{hw(qv)}{2k_BT}\right),$$

where $w(qv)$ is the phonon density of states and the h is Planck's constant.

The reaction barrier is calculated using the constrained energy minimization method. All transition states were estimated using the climbing image nudged elastic band method (CI-NEB)[11]. The adsorption energy(E_{ads}) of molecular CO, N_2, atomic O is calculated according to equations:

$$E_{ads-CO} = E_{slab-CO} - (E_{slab} + E_{CO});$$

$$E_{ads-N_2} = E_{slab-N_2} - (E_{slab} + E_{N_2});$$

$$E_{ads-O} = E_{slab-O} - (E_{slab} + \frac{1}{2}E_{O_2});$$

where $E_{slab-CO}$, E_{ads-N_2}, E_{ads-O}, are the total energies of adsorption models of CO, N_2 and O on the surface, respectively. E_{slab} is the total energy of the bare slab, and E_{CO}, E_{N_2}, E_{O_2} are the total energies of the free adsorbates in the gas phase. Therefore, the more negative the adsorption energy, the stronger the adsorption. The activation energy (or reaction barrier) (E_a) and reaction energy (E_r) are calculated according to equations of

$$E_a = E_{TS} - E_{IS}$$

$$E_r = E_{FS} - E_{IS}$$

where E_{IS}, E_{FS} and E_{TS} are the energies of the corresponding initial state (IS), final state (FS) and transition state (TS), respectively.

(1) G. Kresse and J. Furthmüller, Efficient Iterative Schemes for ab initio Total-Energy Calculations Using a Plane-Wave Basis Set. *Phys. Rev. B,* 1996, **54**, 11169–11186.

(2) G. Kresse and J. Hafner, Ab initio Molecular Dynamics for Liquid Metals. *Phys. Rev. B,* 1993, **47**, 558–561.

(3) J. P. Perdew, K. Burke and M. Ernzerhof, *Phys. Rev. Lett.,* 1996, **77**, 3865–3868.

(4) G. Kresse and J. Joubert, *Phys. Rev. B,* 1999, **59**, 1758–1775.

(5) P. E. Blöchl, *Phys. Rev. B,* 1994, **50**, 17953–17979.

(6) H. J. Monkhorst and J. D. Pack, *Phys. Rev. B,* 1976, **13**, 5188–5192.

(7) J. M. Cowley, *Phys. Rev.*, 1950, **77**, 669.

(8) R. Ferrando, A. Fortunelli and G. Rossi, *Phys. Rev. B,* 2005, **72**, 085449.

(9) T. Ishimoto and M. Koyama, *J. Phys. Chem. Lett.,* 2016, **7**, 736–740.

(10) H. B. Liu, U. Pal, R. Perez and J. A. Ascencio, *J. Phys. Chem B,* 2006, **110**, 5191–5195.

(11) G. Henkelman, B. P. Uberuaga and H. Jónsson, *J. Chem. Phys.,* 2000, **113**, 9901-9904

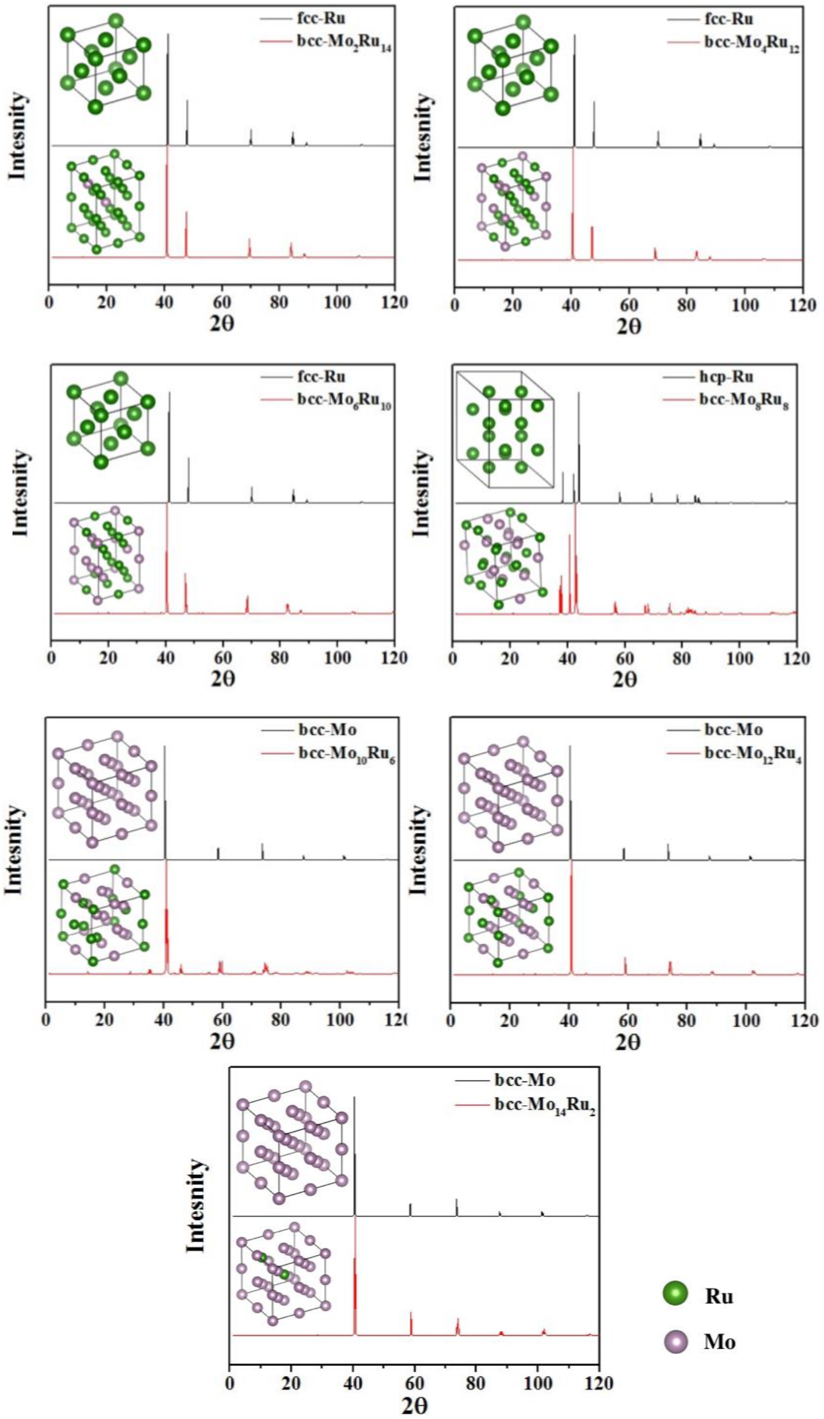

Fig. S1. The structures of optimized bcc-MoRu alloys and their XRD patterns calculated by VESTA. The Warren–Cowley parameter (a_i) values for bcc-Mo_2Ru_{14}, bcc-Mo_4Ru_{12}, bcc-Mo_6Ru_{10}, bcc-Mo_8Ru_8, bcc-$Mo_{10}Ru_6$, bcc-$Mo_{12}Ru_4$, bcc-$Mo_{14}Ru_2$ are 0.333, 0, 0.037, 0, 0.37, 0, 0.333, respectively.

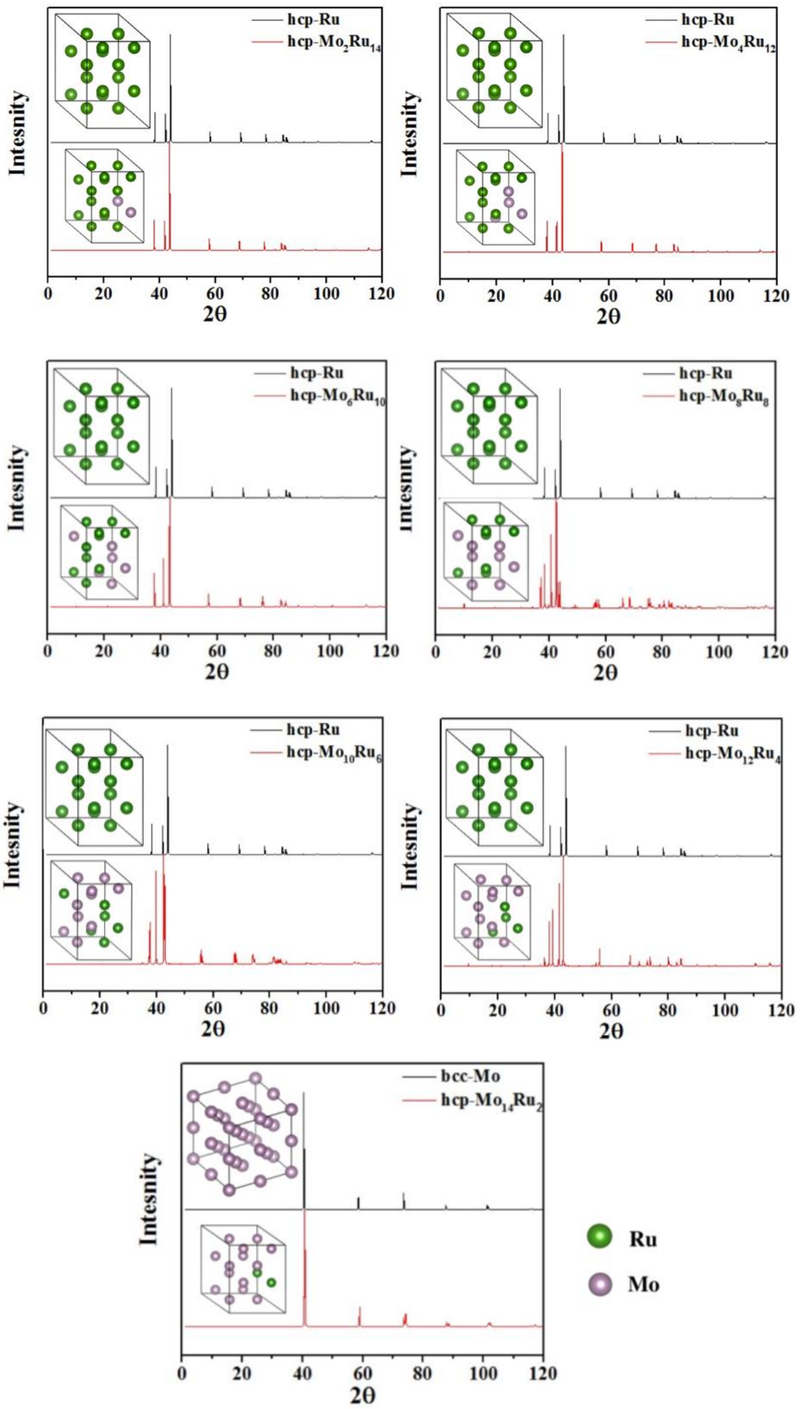

Fig. S2. The structures of optimized hcp-MoRu alloys and their XRD patterns calculated by VESTA. The Warren–Cowley parameter (a_i) values for hcp-Mo_2Ru_{14}, hcp-Mo_4Ru_{12}, hcp-Mo_6Ru_{10}, hcp-Mo_8Ru_8, hcp-$Mo_{10}Ru_6$, hcp-$Mo_{12}Ru_4$, hcp-$Mo_{14}Ru_2$ are 0.333, 0, 0.037, 0, 0.37, 0, 0.333, respectively

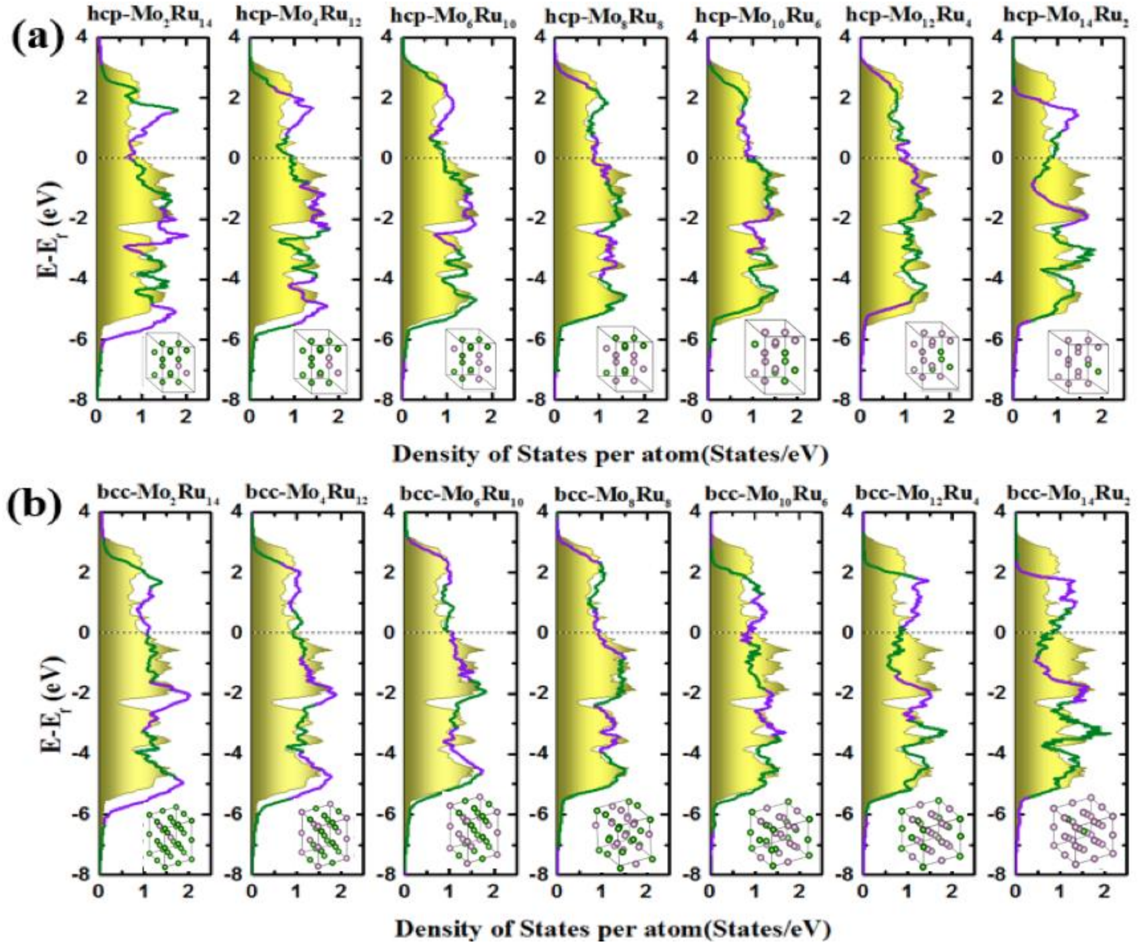

Fig. S3. DOS of Mo–Ru alloy systems compared with that of hcp-Tc_{16}. Bicolor lines are Mo–Ru alloy, yellow patterns are hcp-Tc_{16}. (a) DOS of hcp-MoRu; (b) DOS of bcc-MoRu. For hcp-Mo_2Ru_{14} and hcp-$Mo_{14}Ru_2$, obvious mismatched DOS peaks exist. For hcp-Mo_8Ru_8, there is no obvious mismatched DOS peaks to those of Tc, but some subtle distinctions. This tendency of DOS shape changes proves that the iso-valence electrons are a prerequisite for creating a pseudo-metal. One exception is hcp-$Mo_{10}Ru_6$; in a local range from −2 eV to the Fermi level, its DOS shape and intensity are consistent with those of Tc. Similar results in bcc type alloy of Mo-Ru, bcc-Mo_8Ru_8 is the most similar to Tc.

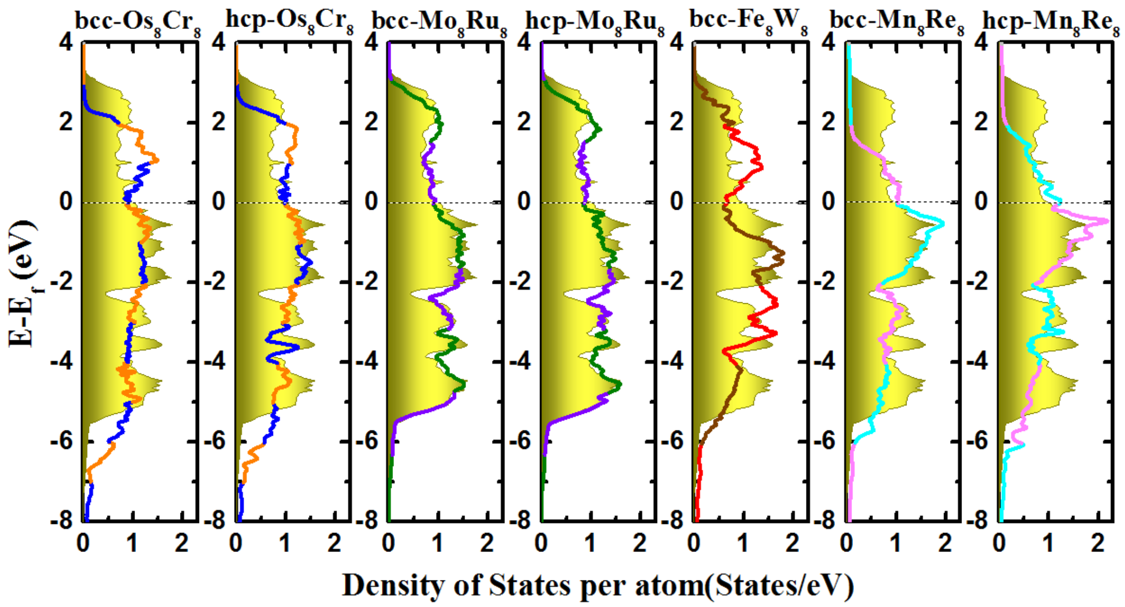

Fig. S4. DOS of bcc-Os_8Cr_8, hcp-Os_8Cr_8, bcc-Mo_8Ru_8, hcp-Mo_8Ru_8, bcc-Fe_8W_8, bcc-Mn_8Re_8, and hcp-Mn_8Re_8 (bicolor lines) compared with that of hcp-Tc_{16} (yellow patterns). All alloys have the same number of valence electrons as the Tc alloy. However, only Mo-Ru alloy has a similar DOS shape to Tc. Neither Os–Cr nor Fe–W alloys have a coincident DOS to that of Tc throughout the whole energy level. Mn–Re alloys have only one part similar to that of Tc, the matched DOS peak located at −0.53 eV. For Mo–Ru alloys, there is no obvious difference or mismatched DOS peak between Mo–Ru and Tc. Mo–Ru alloy is the only candidate suitable for pseudo-Tc.

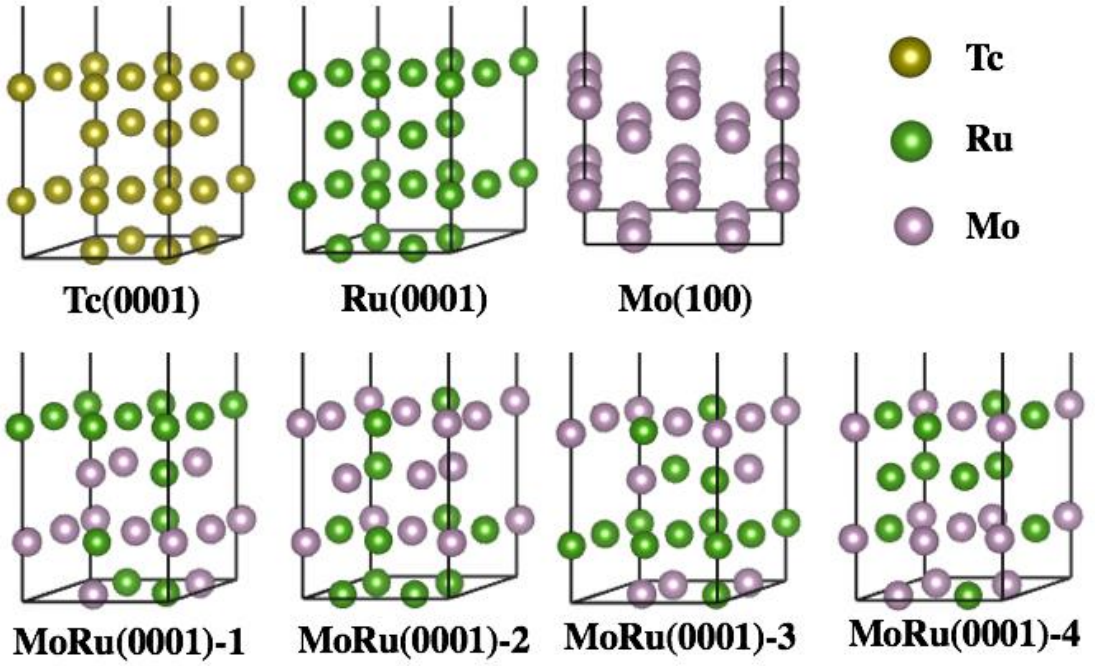

Fig. S5 The slab models of Tc(0001), Ru(0001), Mo(100), MoRu(0001)

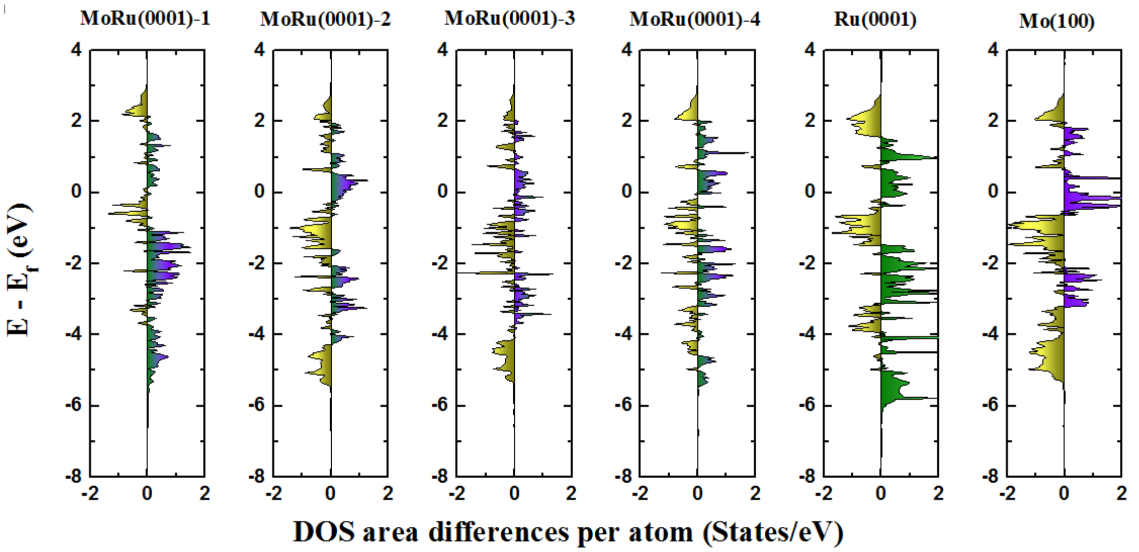

Figure S6 DOS area differences of surface comparing to Tc.

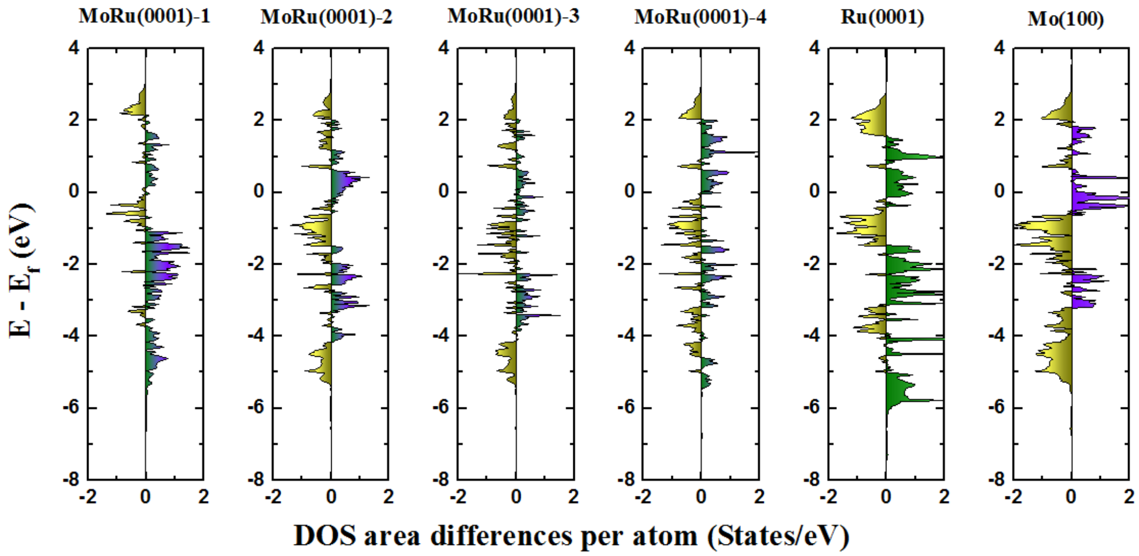

Figure S7 DOS area differences of active site on the surfaces comparing to Tc. MoRu(0001)-1 is fcc site (or hcp site, Ru_3 site); MoRu(0001)-2, MoRu(0001)-3, MoRu(0001)-4 are fcc sites (or hcp sites, Mo_2Ru-site); Ru(0001) is fcc site (or hcp site, Ru_3site), and Mo(100) is hcp site (Mo_4 site).

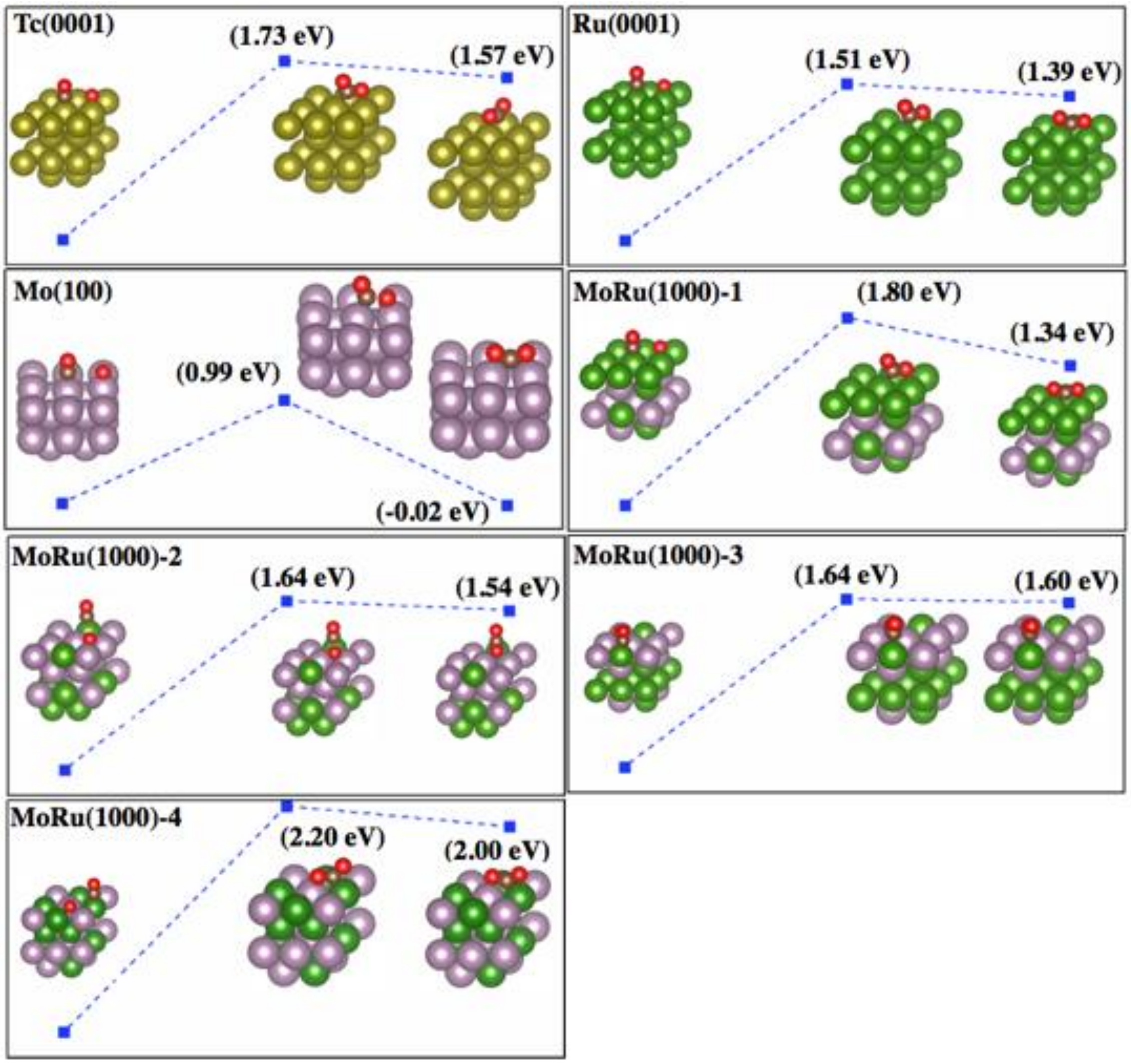

Fig. S8 The details of energy pathways and side views of IS, TS, and FS for CO oxidation on the Tc(0001), Ru(0001), Mo(100), and four MoRu(0001) surfaces. The values are the activation energy (E_a) and the reaction energy (E_r).

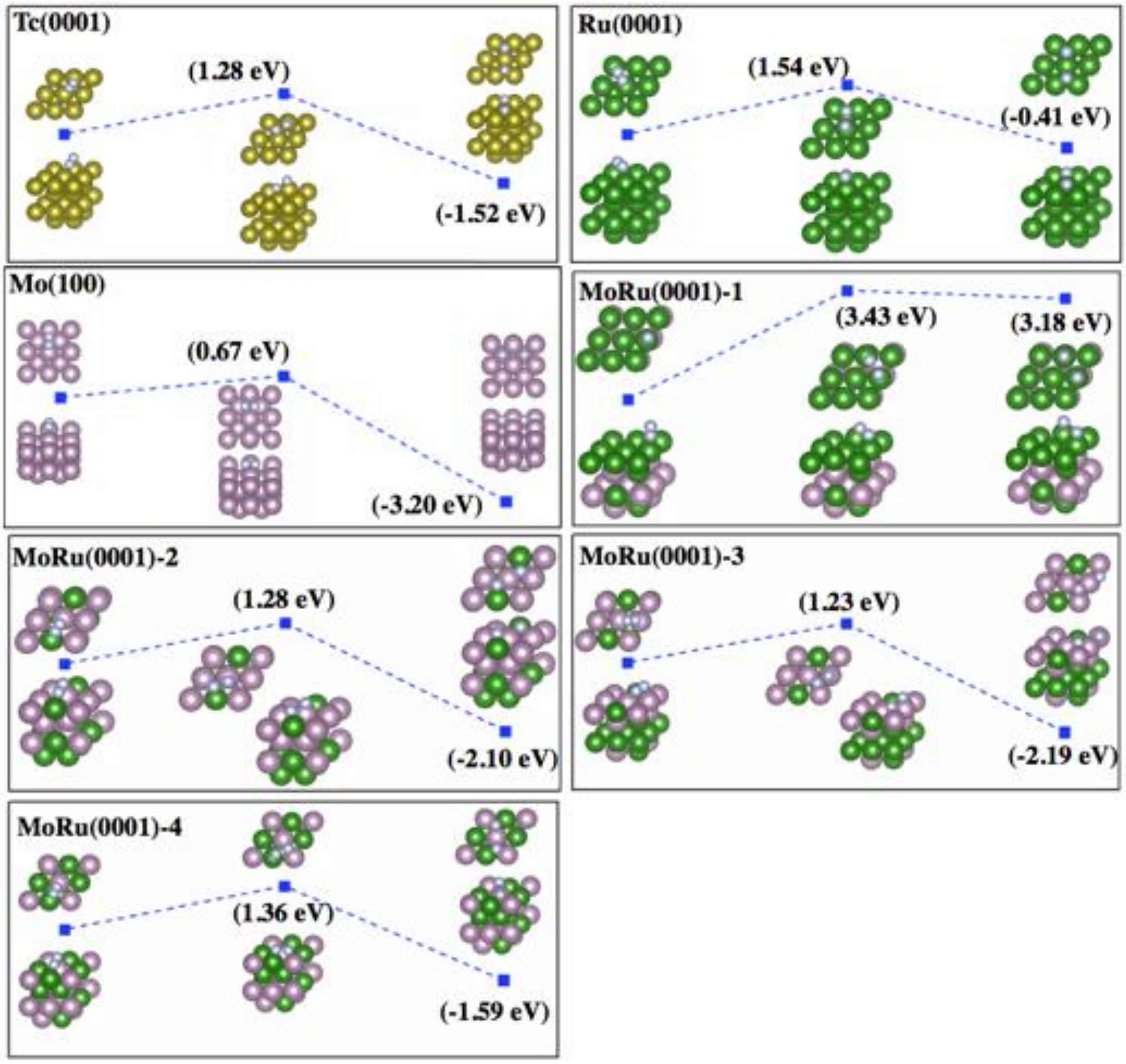

Fig. S9 The details of energy pathways and side views of IS, TS, and FS for N_2 dissociation on the Tc(0001), Ru(0001), Mo(100), and four MoRu(0001) surfaces. The values are the activation energy (E_a) and the reaction energy (E_r).

Table S1. Peak positions of hcp-Mo_8Ru_8, hcp-$Mo_{10}Ru_6$, and bcc-Mo_8Ru_8 against hcp-Tc_{16} in the lower energy range (from negative infinity to the boundary).

	Peak 1		Peak 2		Peak 3	
	Position (eV)	Intensity	Position (eV)	Intensity	Position (eV)	Intensity
hcp-Tc_{16}	−4.47	1.59	−3.57	1.59	−2.97	1.36
hcp-Mo_8Ru_8	−4.57	1.61	−3.42	1.39	−2.77	1.42
hcp-$Mo_{10}Ru_6$	−4.36	1.58	−3.46	1.48	−2.71	1.34
bcc-Mo_8Ru_8	−4.62	1.52	−3.42	1.42	−2.97	1.31

Table S2. Ranges of DOS platform by energy level and the average DOS intensity in these ranges.

	Energy range (eV)	Start position (eV)	End position (eV)	Intensity
hcp-Tc_{16}	1.33	−0.56	−1.89	1.47
hcp-Mo_8Ru_8	1.60	−0.32	−1.92	1.28
hcp-$Mo_{10}Ru_6$	1.30	−0.51	−1.81	1.36
bcc-Mo_8Ru_8	1.10	−0.82	−1.92	1.44

Table S3. Vibration entropy effect for hcp-Mo_8Ru_8 and bcc-Mo_8Ru_8 after correcting with pure hcp-Ru_{16} and bcc-Mo_{16}.

Temperature (K)	hcp-Mo_8Ru_8 (eV/K)	bcc-Mo_8Ru_8 (eV/K)
0	0	0
10	1.04×10^{-6}	6.87×10^{-7}
20	3.92×10^{-6}	2.45×10^{-6}
30	7.8×10^{-6}	4.73×10^{-6}
40	1.13×10^{-5}	6.95×10^{-6}
50	1.38×10^{-5}	8.82×10^{-6}
60	1.54×10^{-5}	1.03×10^{-5}
70	1.63×10^{-5}	1.15×10^{-5}
80	1.68×10^{-5}	1.24×10^{-5}
90	1.71×10^{-5}	1.31×10^{-5}
100	1.72×10^{-5}	1.37×10^{-5}
110	1.71×10^{-5}	1.42×10^{-5}
120	1.7×10^{-5}	1.46×10^{-5}
130	1.69×10^{-5}	1.5×10^{-5}
140	1.67×10^{-5}	1.52×10^{-5}
150	1.65×10^{-5}	1.55×10^{-5}
160	1.63×10^{-5}	1.57×10^{-5}
170	1.61×10^{-5}	1.59×10^{-5}
180	1.59×10^{-5}	1.61×10^{-5}
190	1.57×10^{-5}	1.63×10^{-5}
200	1.55×10^{-5}	1.64×10^{-5}
210	1.52×10^{-5}	1.66×10^{-5}
220	1.5×10^{-5}	1.67×10^{-5}
230	1.48×10^{-5}	1.68×10^{-5}
240	1.47×10^{-5}	1.69×10^{-5}
250	1.45×10^{-5}	1.7×10^{-5}
260	1.43×10^{-5}	1.71×10^{-5}
270	1.41×10^{-5}	1.72×10^{-5}
280	1.39×10^{-5}	1.73×10^{-5}
290	1.38×10^{-5}	1.73×10^{-5}
300	1.36×10^{-5}	1.74×10^{-5}
310	1.34×10^{-5}	1.75×10^{-5}
320	1.33×10^{-5}	1.76×10^{-5}
330	1.31×10^{-5}	1.76×10^{-5}
340	1.3×10^{-5}	1.77×10^{-5}
350	1.28×10^{-5}	1.77×10^{-5}
360	1.27×10^{-5}	1.78×10^{-5}

370	1.25×10^{-5}	1.79×10^{-5}
380	1.24×10^{-5}	1.79×10^{-5}
390	1.23×10^{-5}	1.8×10^{-5}
400	1.21×10^{-5}	1.8×10^{-5}
410	1.2×10^{-5}	1.81×10^{-5}
420	1.19×10^{-5}	1.81×10^{-5}
430	1.18×10^{-5}	1.82×10^{-5}
440	1.17×10^{-5}	1.82×10^{-5}
450	1.15×10^{-5}	1.82×10^{-5}
460	1.14×10^{-5}	1.83×10^{-5}
470	1.13×10^{-5}	1.83×10^{-5}
480	1.12×10^{-5}	1.84×10^{-5}
490	1.11×10^{-5}	1.84×10^{-5}
500	1.1×10^{-5}	1.84×10^{-5}
510	1.09×10^{-5}	1.85×10^{-5}
520	1.08×10^{-5}	1.85×10^{-5}
530	1.07×10^{-5}	1.85×10^{-5}
540	1.06×10^{-5}	1.86×10^{-5}
550	1.05×10^{-5}	1.86×10^{-5}
560	1.04×10^{-5}	1.86×10^{-5}
570	1.03×10^{-5}	1.87×10^{-5}
580	1.02×10^{-5}	1.87×10^{-5}
590	1.01×10^{-5}	1.87×10^{-5}
600	1×10^{-5}	1.88×10^{-5}
610	9.94×10^{-6}	1.88×10^{-5}
620	9.85×10^{-6}	1.88×10^{-5}
630	9.76×10^{-6}	1.89×10^{-5}
640	9.68×10^{-6}	1.89×10^{-5}
650	9.6×10^{-6}	1.89×10^{-5}
660	9.52×10^{-6}	1.89×10^{-5}
670	9.44×10^{-6}	1.9×10^{-5}
680	9.36×10^{-6}	1.9×10^{-5}
690	9.28×10^{-6}	1.9×10^{-5}
700	9.2×10^{-6}	1.9×10^{-5}
710	9.13×10^{-6}	1.91×10^{-5}
720	9.05×10^{-6}	1.91×10^{-5}
730	8.98×10^{-6}	1.91×10^{-5}
740	8.9×10^{-6}	1.91×10^{-5}
750	8.83×10^{-6}	1.92×10^{-5}
760	8.76×10^{-6}	1.92×10^{-5}
770	8.69×10^{-6}	1.92×10^{-5}
780	8.62×10^{-6}	1.92×10^{-5}

790	8.55×10^{-6}	1.92×10^{-5}
800	8.48×10^{-6}	1.93×10^{-5}
810	8.42×10^{-6}	1.93×10^{-5}
820	8.35×10^{-6}	1.93×10^{-5}
830	8.29×10^{-6}	1.93×10^{-5}
840	8.22×10^{-6}	1.94×10^{-5}
850	8.16×10^{-6}	1.94×10^{-5}
860	8.1×10^{-6}	1.94×10^{-5}
870	8.03×10^{-6}	1.94×10^{-5}
880	7.97×10^{-6}	1.94×10^{-5}
890	7.91×10^{-6}	1.95×10^{-5}
900	7.85×10^{-6}	1.95×10^{-5}
910	7.79×10^{-6}	1.95×10^{-5}
920	7.73×10^{-6}	1.95×10^{-5}
930	7.67×10^{-6}	1.95×10^{-5}
940	7.62×10^{-6}	1.95×10^{-5}
950	7.56×10^{-6}	1.96×10^{-5}
960	7.5×10^{-6}	1.96×10^{-5}
970	7.45×10^{-6}	1.96×10^{-5}
980	7.39×10^{-6}	1.96×10^{-5}
990	7.34×10^{-6}	1.96×10^{-5}
1000	7.28×10^{-6}	1.96×10^{-5}

Table S4 The adsorption structures and energies of molecule CO and atomic O on the surfaces of Tc(0001), Ru(0001), Mo(100).

CO	Top	fcc or bridge	hcp
Tc(0001)	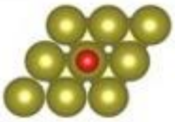 E_{ads}= -1.72 eV	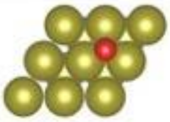 E_{ads}= -1.80 eV	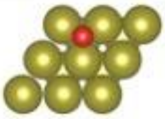 E_{ads}= -1.60 eV
Ru(0001)	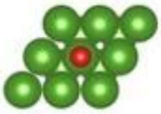 E_{ads}= -1.91 eV	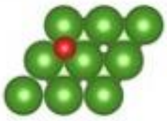 E_{ads}= -1.75 eV	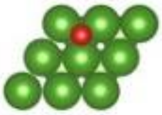 E_{ads}= -1.88 eV
Mo(100)	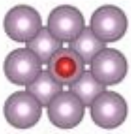 E_{ads}= -1.73 eV	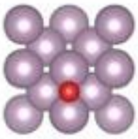 E_{ads}= -1.80 eV	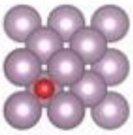 E_{ads}= -2.21 eV

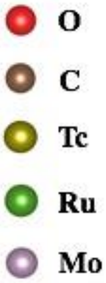

O	Top	fcc or bridge	hcp
Tc(0001)	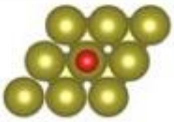 E_{ads}= -2.23 eV	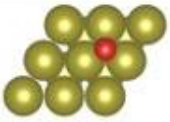 E_{ads}= -3.01 eV	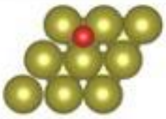 E_{ads}= -3.67 eV
Ru(0001)	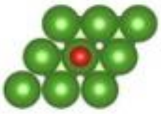 E_{ads}= -1.47 eV	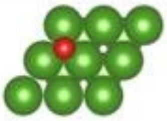 E_{ads}= -2.45 eV	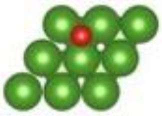 E_{ads}= -2.90 eV
Mo(100)	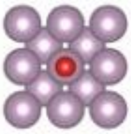 E_{ads}= -3.39 eV	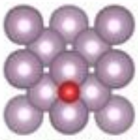 E_{ads}= -3.93 eV	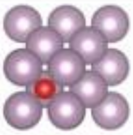 E_{ads}= -4.08 eV

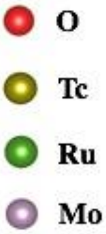

Table S5 The adsorption structures and energies of molecular CO on MoRu(0001). All the possible adsorption sites have been considered, only stable structures are listed.

RuMo(0001)-1	Bridge	Ru-top	fcc
	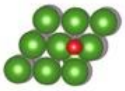 E_{ads}= -1.43 eV	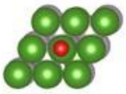 E_{ads}= -1.64 eV	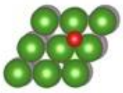 E_{ads}= -1.61 eV
	Mo-hcp	Ru-hcp	
	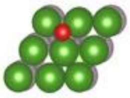 E_{ads}= -1.61 eV	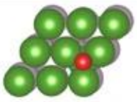 E_{ads}= -1.50 eV	

RuMo(0001)-2	Bridge	Mo_2Ru-hcp	Mo_3-fcc	
	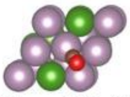 E_{ads}= -1.84 eV	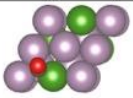 E_{ads}= -2.04 eV	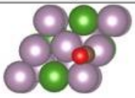 E_{ads}= -1.89 eV	
	Mo_2Ru-fcc	MoRu-bridge	Ru-top	Mo-top
	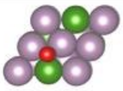 E_{ads}= -2.01 eV	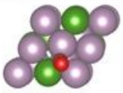 E_{ads}= -1.90 eV	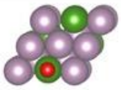 E_{ads}= -1.96 eV	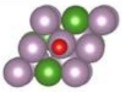 E_{ads}= -1.73 eV

RuMo(0001)-3	Mo_2Ru-fcc	Mo_3-fcc	Mo_3-hcp	
	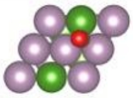 E_{ads}= -2.01 eV	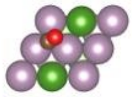 E_{ads}= -1.95 eV	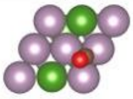 E_{ads}= -1.84 eV	
	Mo_2Ru-hcp	Mo_2Ru-hcp	Ru-top	Mo-top
	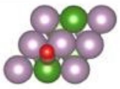 E_{ads}= -1.99 eV	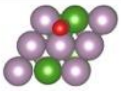 E_{ads}= -1.79 eV	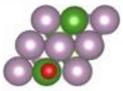 E_{ads}= -1.95 eV	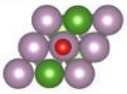 E_{ads}= -1.70 eV

RuMo(0001)-4	RuRu-bridge	RuRu-bridge	Mo-top
	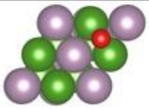 E_{ads}= -1.95 eV	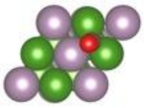 E_{ads}= -1.98 eV	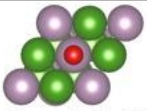 E_{ads}= -1.59 eV
	Ru-top	Ru-top	Ru-top
	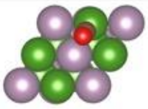 E_{ads}= -1.99 eV	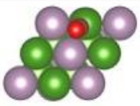 E_{ads}= -2.01 eV	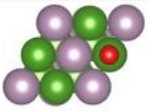 E_{ads}= -2.08 eV

Table S6 The adsorption structures and energies of atomic O on MoRu(0001). All the possible adsorption sites have been considered, only stable structures are listed.

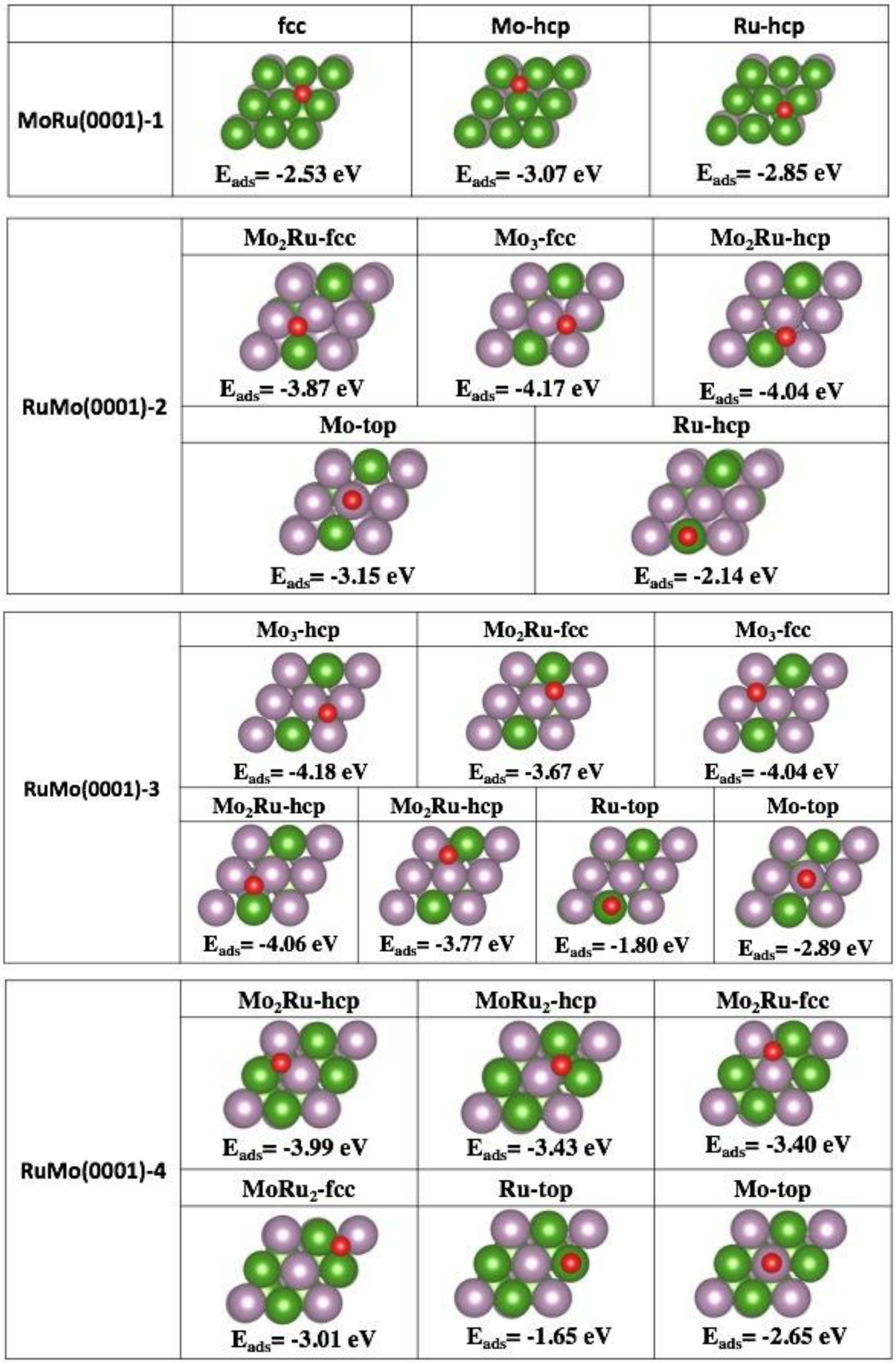

Table S7 The adsorption structures and energies of molecular O on Tc(0001), Ru(0001), Mo(100), and four MoRu(0001) surface. All the possible bridge adsorption sites have been considered. The red value means this adsorption structure was used as IS for TS search.

N_2	Tc(0001)	Ru(0001)	Mo(100)
**Pure Surface**	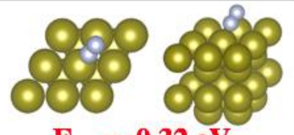 E_{ads}= -0.32 eV	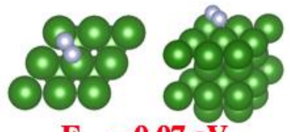 E_{ads}= 0.07 eV	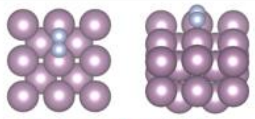 E_{ads}= -0.91 eV
**MoRu(0001)-1**	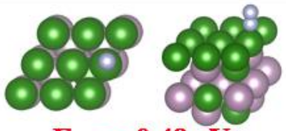 E_{ads}= -0.49 eV		
**MoRu(0001)-2**	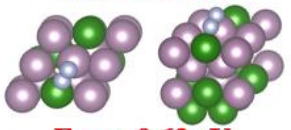 E_{ads}= -0.62 eV	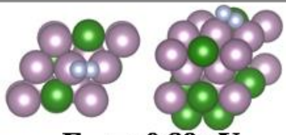 E_{ads}= -0.89 eV	
**MoRu(0001)-3**	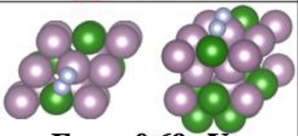 E_{ads}= -0.69 eV	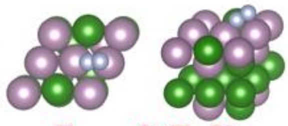 E_{ads}= -0.67 eV	
**MoRu(0001)-4**	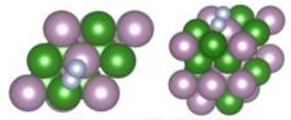 E_{ads}= -0.39 eV	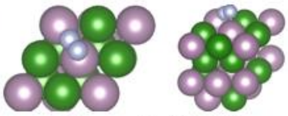 E_{ads}= -0.48 eV	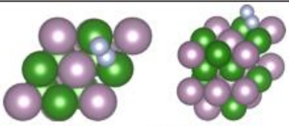 E_{ads}= -0.36 eV
